# A Recurrent *FAM83H* Mutation in an Extended Colombian Family and Variable Craniofacial Phenotypes

**DOI:** 10.3390/children9030362

**Published:** 2022-03-04

**Authors:** Camila Alvarez, María Andrea Aragón, Yejin Lee, Sandra Gutiérrez, Patricia Méndez, Dabeiba Adriana García, Liliana Otero, Jung-Wook Kim

**Affiliations:** 1 Pediatric Dentist Residency Program, Faculty of Dentistry, Pontifical Xavierian University, Bogota 110231, Colombia; camila-alvarez@javeriana.edu.co (C.A.); aragon-m@javeriana.edu.co (M.A.A.); mendezp@javeriana.edu.co (P.M.); 2 Department of Pediatric Dentistry, School of Dentistry & DRI, Seoul National University, Seoul 03080, Korea; lyj72255621@gmail.com; 3 Center of Dental Research, Pontifical Xavierian University, Bogota 110231, Colombia; s.gutierrez@javeriana.edu.co (S.G.); garciad@javeriana.edu.co (D.A.G.); 4 Department of Molecular Genetics, School of Dentistry & DRI, Seoul National University, Seoul 03080, Korea

**Keywords:** hereditary enamel defects, *FAM83H*, hypocalcified, anterior open bite, tooth impaction, crown resorption

## Abstract

Amelogenesis imperfecta (AI) is a collection of rare genetic disorders affecting the quantity and/or quality of the tooth enamel. AI can be classified into three major types according to the clinical phenotype: hypoplastic, hypocalcified, and hypomatured. Among them, the hypocalcified type shows the weakest physical properties, leaving rough and discolored enamel surfaces after tooth eruption. To date, mutations in the *FAM83H* gene are responsible for the autosomal-dominant hypocalcified AI. In this study, we recruited a four-generation Colombian family with hypocalcified AI and identified a recurrent nonsense mutation in the *FAM83H* gene (NM_198488.5:c.1289C>A, p.(Ser430 *)) by candidate gene sequencing. Cephalometric analyses revealed the anterior open bite that occurred in the proband is not correlated with the AI in this family.

## 1. Introduction

Amelogenesis imperfecta (AI) is a rare genetic disorder affecting the tooth enamel [1]. AI is heterogeneous in terms of etiology and clinical phenotype. To date, more than 20 genes are known to be involved in the molecular pathogenesis of AI [2,3]. Clinically, AI can be classified as one or mixed types of hypoplastic, hypomature, and hypocalcified enamel [4]. Various surface characteristics make it even more heterogeneous.

*FAM83H* mutations have been found to cause autosomal-dominant hypocalcified AI (ADHCAI) [5,6]. Other candidate genes for ADHCAI have not been discovered. *FAM83H* mutations causing ADHCAI are nonsense and frameshift mutations in the last exon, which generate truncated proteins that escape from nonsense-mediated mRNA decay [7]. Interestingly, the normal *FAM83H* is shown to be dispensable for proper enamel formation [8]. The truncated protein translocates into the nucleus, while its wild-type counterpart locates into the cytoplasm [9]. Despite the fact that the functional role of *FAM83H* remains to be elucidated, it has been demonstrated with a mouse model that the mutation mechanism causing ADHCAI is neomorphic [10].

In this study, we recruited a four-generation Colombian family with hypocalcified AI and sequenced the *FAM83H* gene to identify a disease-causing mutation. Cephalometric analyses were performed to characterize whether there is a common craniofacial skeletal feature among the affected family members in the family.

## 2. Materials and Methods

We recruited an extended Colombian AI family. The study protocol was reviewed and approved by the institutional review board of the Pontificia Universidad Javeriana and the Seoul National University Dental Hospital. Informed consent was obtained from all subjects participating in this study with an understanding of the nature of the study.

The family history was investigated to build the pedigree. Clinical examinations were performed for the participating family members to identify and characterize the dental phenotype. Panoramic and lateral cephalogram radiographs were taken from the participating family members. After tracing the cephalograms with Dolphin^®^ software version 11.9 (Dolphin Imaging & Management Solutions, Chatsworth, CA, USA), the measurement standards were analyzed to establish the skeletal and facial phenotype.

Saliva samples were collected for the mutational analysis using Oragene^®^ DNA saliva collection kits (DNA Genotek Inc., Ottawa, ON, Canada). Genomic DNA was isolated from saliva samples with the conventional salting-out method and the quantity and quality were measured. Candidate gene sequencing for the *FAM83H* gene was performed with a DNA sample of the proband as previously described [5]. The segregation of the identified mutation within the family was investigated by Sanger sequencing. Sanger DNA sequencing was performed using ABI 3730xl DNA Analyzer (Applied Biosystems, Foster City, CA, USA) at the DNA sequencing center (Macrogen, Seoul, Korea).

## 3. Results

**Figure 1:**
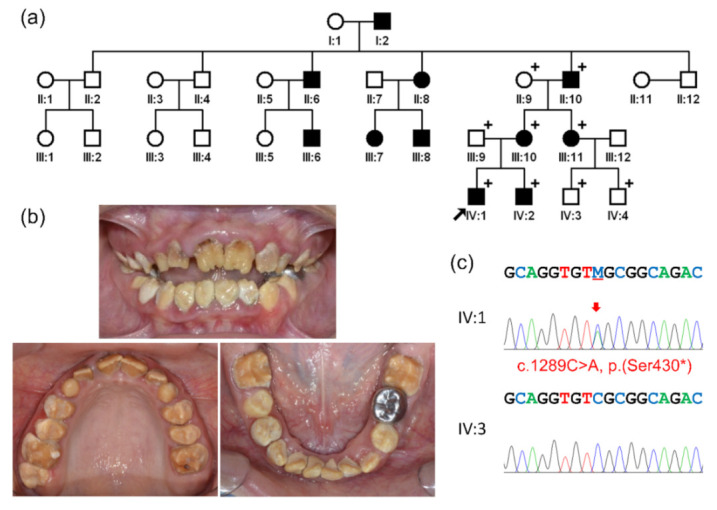
Pedigree, clinical photos and sequencing chromatograms: (**a**) pedigree of the family. Affected family members are indicated by black-filled symbols and the participating members are indicated with a plus sign (+) at the upper right corner of the symbols. The black arrow indicates the proband. (**b**) Clinical photos of the proband at age 10 years 6 months. A brown-discolored, rough enamel surface can be seen in all teeth. Heavy calculus deposits and an anterior open bite can be observed. (**c**) Sequencing chromatograms are shown for the proband (IV:1) and an unaffected cousin (IV:3). The mutation is indicated by the red arrow (M: C and A).

In this study, we recruited a four-generation Colombian AI family (Figure 1). A pedigree analysis strongly suggested an autosomal dominant inheritance pattern. No family member reported other remarkable past medical history, including genetic abnormalities or systemic diseases. Radiographically, the erupted teeth presented thin enamel coverage by breakdown but the unerupted developing teeth showed enamel with normal thickness but reduced radiopacity, being similar to the dentin. The affected enamel was very weak and easily worn, leaving brown-discolored, rough surfaces, suggesting hypocalcified AI. Therefore, mutational screening for the *FAM83H* gene was performed as a candidate gene approach.

Sanger sequencing of the exons and exon/intron boundaries of all five exons of *FAM83H* revealed a nonsense mutation in the last exon. The mutation was a transversion change of cytosine to adenine (NM_198488.5:c.1289C>A), changing serine (TCG) to an amber stop codon (TAG) at amino acid position 430 (NP_940890.4:p.(Ser430 *)). The mutation would produce a truncated protein of 429 amino acids instead of 1179 wild-type FAM83H. Perfect segregation of the mutation and the disease phenotype in the family was confirmed. This is the first *FAM83H* mutation identified in the Colombian population.

Clinically, the proband (IV:1) presented an anterior open bite, unlike his younger brother (IV:2), who showed a deep bite. The cephalometric analysis of proband revealed skeletal Class III malocclusion and skeletal open bite. The other affected individuals (III:11 and IV:2) presented skeletal Class I malocclusion as well as straight profile.

## 4. Discussion

The mutation identified in this study was initially reported in genotype-phenotype studies involving 71 AI families and the affected ADHCAI family was African-American (personal communication with Dr. John Timothy Wright) [11]. Later, a sporadic case was reported in a European study of a cohort of 101 unrelated patients from France, Germany, and Morocco [12]. The family in this study is Mestizo Colombian, suggesting that this mutation is a mutational hotspot in the *FAM83H* gene.

The *FAM83H* gene consists of five exons. The translation start site is located in exon 2 and the last exon encodes most of the amino acids (933 out of 1179 amino acids). The *FAM83H* mutations causing ADHCAI clustered in the anterior half of exon 5. Therefore, the truncated proteins without a specific sequence in the exon 5, lose their normal cytoplasmic localization but translocate to the nucleus and hamper normal enamel calcification process [7].

**Figure 2:**
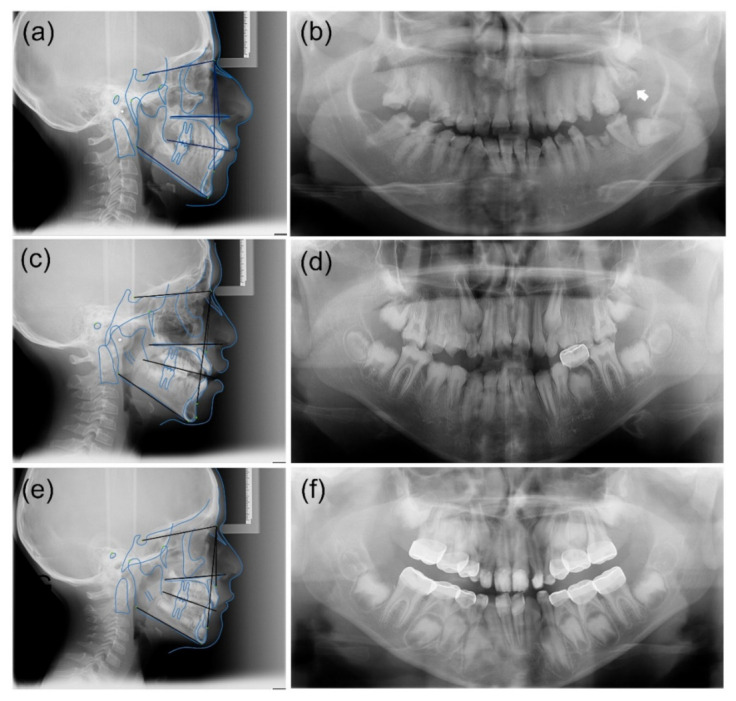
Cephalometric and panoramic radiographs of the affected individuals. (**a**,**b**) Cephalometric and panoramic radiographs of an aunt of the proband (III:11) at age 29 years 10 months. Impactions of the mandibular left third molar and maxillary left second and third molars were identified. Crown resorption (white arrow) of the impacted maxillary left second molar was found to have occurred. The anterior overbite appears to be within the normal range. (**c**,**d**) Cephalometric and panoramic radiographs of the proband (IV:1) at age 10 years 6 months. Hypocalcified enamel with reduced radiodensity is clearly observable in the developing second and third molars. The proband has an anterior open bite. (**e**,**f**) Cephalometric and panoramic radiographs of a younger brother of the proband (IV:2) at age 8 years 8 months. Multiple restorations were performed with a stainless-steel crown and resin composite. Anterior teeth tended toward mild to moderately deep overbite.

The open bite tendency has been identified in AI patients regardless of their molecular etiology [13,14]. Some, but not all, ADHCAI families also show an association of the open bite or Angle’s Class III malocclusion [5,6]. The proband in this study had an anterior open bite. To find the association between the malocclusion and the identified *FAM83H* mutation, cephalometric analyses were conducted on several affected individuals (III:11, IV:1, and IV:2). As a result, the anterior open bite was not consistent among the affected individuals, despite the fact that their skeletal characteristics did not differ considerably (Figure 2, Table 1). Slightly increased overjet was identified in a younger brother of the proband. Therefore, it appears that the anterior open bite is not associated with the *FAM83H* mutation itself, at least in this family.

Tooth impaction is most frequently related to the enamel renal syndrome (ERS, OMIM #204690) [15,16,17]. This is caused by recessive *FAM20A* mutations, characterized by hypoplastic AI, pulp stones, delayed or failed eruptions of secondary dentition, gingival overgrowth, and nephrocalcinosis. In addition to tooth impaction caused by *FAM20A* mutations, impaction and/or crown resorption have been reported in the other non-syndromic AI patients [7,18]. Specifically, hypoplastic and hypocalcified AI are more frequently linked to this phenotype compared to hypomature AI. Tooth eruption could be less efficient with a rough enamel surface than a smooth and well-mineralized enamel surface. Furthermore, a rough and less-mineralized enamel surface could be prone to resorption when impaction occurs. In this study, impaction and crown resorption of the left maxillary second molar were noted in an affected individual (III:11). Routine follow-up and early intervention, such as surgical window opening, could prevent extraction caused by impaction and crown resorption.

In conclusion, in this report, we present a recurrent *FAM83H* mutation as a candidate gene approach, for the first time, in an extended ADHCAI Colombian family. Impaction and crown resorption were observed in an affected individual, supporting the previous finding. The proband had an anterior open bite, but it was not correlated with the disease in this family. This finding will improve our understanding of the genotype–phenotype relationship in ADHCAI caused by the *FAM83H* mutation.

## Figures and Tables

**Table 1:** Cephalometric Measurements of the Affected Individuals.

Measurements	Normal Values	III:11	IV:1	IV:2
SNA	82° ± 2°	84°	82°	83°
SNB	80° ± 2°	80°	75°	77°
ANB	2° ± 2°	3°	7°	6°
A to N perp	−2.0 ± 3.7 mm	2 mm	7 mm	−7 mm
B to N perp	−6.9 ± 4.3 mm	−11 mm	−15 mm	−13 mm
A-N	0.4 ± 1 mm	1 mm	4 mm	3 mm
Pg-N	−1.8 ± 4.5 mm	−6 mm	−11 mm	−9 mm
N-A-Pg	4° ± 6.4°	7°	8°	6°
SN-PM	32°	38°	40°	32°
U1-SN	103.9° ± 5.5°	112°	111.5°	99.5°
L1-PM	95° ± 7°	93°	101.2°	84.2°
PP-PM	25°	35°	33°	32°

Abbreviations: S = Sella; N = Nasion; A = A-point; B = B-point; Pg = Pogonion; Nperp = Nasion perpendicular line; PM = mandibular plane; U1 = axis of the upper incisor; L1 = axis of the upper incisor; and PP = palatal plane.
